# The Neuroblastoma Microenvironment, Heterogeneity and Immunotherapeutic Approaches

**DOI:** 10.3390/cancers16101863

**Published:** 2024-05-13

**Authors:** Panagiotis Alkinoos Polychronopoulos, Oscar C. Bedoya-Reina, John Inge Johnsen

**Affiliations:** 1 Childhood Cancer Research Unit, Department of Women’s and Children’s Health, Karolinska Institutet, 11883 Stockholm, Sweden; alkinoos.polychronopoulos@ki.se (P.A.P.); oscar.bedoya.reina@ki.se (O.C.B.-R.); 2 School of Medical Sciences, Örebro University, 70182 Örebro, Sweden

**Keywords:** neuroblastoma, tumor microenvironment, neural crest, immunotherapy

## Abstract

**Simple Summary:**

Neuroblastoma, a cancer of the peripheral nervous system, is the most common and deadly tumor that occurs in childhood. In order to cure more patients with neuroblastoma, alternative treatment approaches based on biological understanding of the disease are fundamental. In this review, we discuss the recent developments in neuroblastoma research, with emphasis on new molecular technique approaches for understanding the molecular and cellular interactions within the tumor microenvironment and the development of new treatment modalities holding promise for future treatment regimens.

**Abstract:**

Neuroblastoma is a peripheral nervous system tumor that almost exclusively occurs in young children. Although intensified treatment modalities have led to increased patient survival, the prognosis for patients with high-risk disease is still around 50%, signifying neuroblastoma as a leading cause of cancer-related deaths in children. Neuroblastoma is an embryonal tumor and is shaped by its origin from cells within the neural crest. Hence, neuroblastoma usually presents with a low mutational burden and is, in the majority of cases, driven by epigenetically deregulated transcription networks. The recent development of Omic techniques has given us detailed knowledge of neuroblastoma evolution, heterogeneity, and plasticity, as well as intra- and intercellular molecular communication networks within the neuroblastoma microenvironment. Here, we discuss the potential of these recent discoveries with emphasis on new treatment modalities, including immunotherapies which hold promise for better future treatment regimens.

## 1. Introduction

Neuroblastoma is the most prevalent and lethal cancer that occurs in infancy. It arises predominantly in young children, with a median age of 18 months at diagnosis. Almost 1 in 10 children with cancer suffer from neuroblastoma, and 90% of patients with neuroblastoma are younger than 5 years of age [1,2,3,4]. Neuroblastoma originates from neural crest cells, and primary neoplastic growth is manifested within the loci of the sympathetic nervous system. The most common primary tumor location site is the adrenal medulla, contributing to 47% of cases, whereas 24% are found in the abdominal region, 15% in the thorax region, and 3% in the pelvis and neck [5,6].

The clinical symptoms are variable and may include pain in the abdomen, breathing discomfort, and neurological symptoms. Patients presenting with metastatic disease exhibit weight loss, bone pain, and fever at the time of diagnosis. Clinical syndromes associated with neuroblastoma entail Horner, Pepper, and Hutchinson syndromes [1,4]. Neuroblastoma patients are commonly diagnosed via biopsy, imaging, and levels of vanillylmandelic (VMA) and homovanillic acid (HVA) in the urine, as well as bone marrow aspirate [1,7,8].

Histological examination of neuroblastoma unveils three subtypes according to the degree of neuroblastic differentiation: undifferentiated, poorly differentiated (less than 5% of differentiating neuroblasts), and differentiating tumors (more than 5% of differentiating neuroblasts). According to the International Neuroblastoma Pathology Classification, a favorable or unfavorable prognosis can be determined based on the age of the patient, degree of differentiation, and the mitosis-karyorrhexis index (MKI) [9,10].

In addition, neuroblastoma cases are classified into stages based solely on image-defined risk factors (IDRFs) and patient clinical status. According to the International Neuroblastoma Risk Group Staging System (INRGSS), localized neuroblastoma can be stratified based on the presence (L2) or lack (L1) of IDRFs, while metastatic disease stages distinguish between patients younger than 18 months with skin, liver, or bone marrow metastasis (MS) and all other patients exhibiting metastasis (M) [11]. This classification system allocates patients to five pretreatment risk groups: high-, intermediate-, low- and very low-risk groups, based on prognostic factors and molecular and histological characteristics [12].

## 2. Neuroblastoma Heterogeneity and Origin

Classification in risk groups is also indicative of the clinical tumor heterogeneity observed in neuroblastoma patients and aids the designing and assignment of therapy. Neuroblastoma outcomes can range from non-threatening, very low-risk, and spontaneously regressing tumors to relapsed, treatment-refractory, or metastatic high-risk disease [2,13]. This heterogeneity in clinical outcome is closely linked to the inter- and intratumoral heterogeneity of neuroblastoma [14]. Intertumoral heterogeneity is formed from substantial differences among patients with shared tumor histology, which complicates therapy standardization for neuroblastoma patients. Genetic and epigenetic factors influence intertumoral heterogeneity. On the other hand, intratumoral heterogeneity refers to distinct cell populations within the same tumor and is modulated by multiple factors.

Despite the low mutational burden of pediatric tumors, including neuroblastoma, intratumoral heterogeneity has been identified [15]. At the cellular level, initial studies based on biochemical and morphological features identified three types of cells—neuronal (N-type), substrate (S-type), and intermediate (I-type) cells—in both tumors and cell lines. While S-type cells were non-malignant, N-type cells were able to differentiate between neuronal or neuroendocrine cells and gave rise to tumors in mice. I-type cells were the most malignant of the three and could give rise to either N- or S-type cells [16]. In later studies, tumor cells displaying mesenchymal (MES) and adrenergic (ADRN) identity were identified based on molecular features and epigenetic profiling. These cellular identities may coexist within the same tumor and exhibit the ability to transition between one another in vitro [17,18,19]. Tumor-specific super-enhancer profile analysis has led to sub-classifying ADRN cells into *MYCN* non-amplified low-risk, *MYCN* non-amplified high-risk, and *MYCN*-amplified categories. MES cell phenotypic signature exhibited similarities to Schwann cell precursor cells, a cell type that arises during normal neural crest development and contributes to the formation of the peripheral sympathetic nervous system [20]. Additional evidence from Olsen et al. points to malignant Schwann cell precursor-like cells in neuroblastoma tumors as a cell type facilitating the shift between mesenchymal and adrenergic cell populations [18].

**Figure 1:**
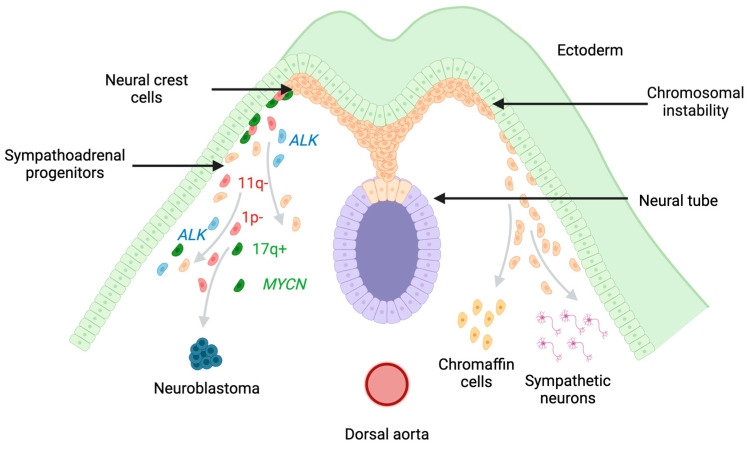
Development of neuroblastoma from the neural crest. Neural crest cells undergo a migra-tion and differentiation process during embryogenesis, giving rise to multiple cell types. Among these are sympathoadrenal progenitor cells, which ultimately differentiate to sympathetic neurons and chromaffin cells. The deregulation of this differentiation process via germline mutations such as the ones on the anaplastic lymphoma kinase (*ALK*) gene or sporadic genetic aberrations like gain of 17q and loss of 1p and 11q, as well as MYCN amplification and others, can give rise to neuroblast-toma. Created with https://www.biorender.com/ (accessed on 7 April 2024).

To understand the heterogeneity in neuroblastoma, uncovering the origin of the disease is imperative. With the majority of primary tumors located in the adrenal medulla and the sympathetic ganglia, taking into account the patients’ clinical picture and tumor-occurrence timing, neuroblastoma is likely to arise from sympathoadrenal progenitor cells of the neural crest lineage [21,22]. During vertebrate development, multipotent neural crest cell precursors emerge between the neural plate and neighboring ectoderm. Following neurulation, these cells migrate to a region in the dorsal neural tube and undergo epithelial-to-mesenchymal transition (EMT), which enables them to delaminate from the neural tube and follow diverse migratory paths [23]. Depending on their location on the anterior–posterior axis, neural crest (NC) cell groups include cranial, cardiac, vagal, and trunk NC cells. The latter will follow three different migratory pathways, giving rise to multiple cell types. Trunk NC cells migrating to the dorsal aorta are predecessors of the sympathoadrenal lineage and will differentiate into sympathetic ganglia and chromaffin cells [24,25] (Figure 1). In the adrenal gland and the sympathetic nervous system, chromaffin cells are endocrine-like cells producing catecholamines [24]. Ambiguity in marker expression has led to the identification of either chromaffin cell phenotypes or sympathoblast transcriptional signatures in neuroblastoma [23,26], further elucidating the sympathoadrenal origin of neuroblastoma. Progenitors of both chromaffin cells and sympathoblasts, such as trunk NC cell-derived Schwann cell precursor cells (SCPs), have been proposed as a potential neuroblastoma cell-of-origin due to their multipotent nature and plasticity [18,27,28].

Neuroblastoma tumor microenvironment (TME) is another crucial factor of heterogeneity. Tumor cells have been shown to interact with stromal cell populations affecting their survival, growth, transcriptomic profile, and metastatic abilities [29,30]. Cellular populations of the neuroblastoma microenvironment include immune cells such as dendritic cells (DCs), tumor-associated macrophages (TAMs), T and B lymphocytes, and natural killer (NK) and natural killer T-cells (NKTs), as well as non-immune cells like cancer-associated fibroblasts (CAFs), Schwann cells (SCs), and mesenchymal stem cells (MSCs) [14,31].

In addition to immune cells, which are discussed in detail below, other non-tumorigenic cells within the neuroblastoma TME have been shown to be important for establishing, maintaining, and fueling the tumorigenic niche. Cancer-associated fibroblasts (CAFs) are a fibroblast-derived population of mesenchymal cells, expressing fibroblast markers such as FAP-a, FSP-1, and more specific markers, such as TN-C, PDPN, and NG2 [32,33]. CAF abundance in neuroblastoma is linked to factors like TGFβ signaling, Schwann cells, and cytokines secreted by tumor-associated macrophages (TAMs) [34,35,36]. Some recently identified CAFs share common characteristics with MSCs, such as CD105, CD90, and CD73, and favor neuroblastoma resistance to chemotherapy by producing inflammatory chemokines and cytokines [29,37,38]. Also, high-risk neuroblastoma with deletion of chromosome 11q exhibits elevated levels of the proinflammatory lipid mediator prostaglandin E2 (PGE2), derived from CAFs expressing high levels of microsomal prostaglandin synthase-1 (mPGES-1) [39]. Small molecule inhibitors of mPGES-1 suppress neuroblastoma growth in preclinical in vivo models, suggesting an important role of CAFs in maintaining the tumor niche in this subgroup of neuroblastoma [40]. Moreover, CAFs have also been shown to promote metastasis by stimulating angiogenesis through VEGF secretion and altering the ECM with collagen production [29]. Additionally, they complicate immunotherapy effectiveness by creating a dense ECM acting as a physical barrier for infiltrating immune cells [41]. Mesenchymal stem cells from various tissues migrate to neuroblastoma tumor sites, lose their multipotency, and contribute to the TME [41,42]. Debates on whether MSCs promote or suppress neuroblastoma are still ongoing. MSCs seem to aid neuroblastoma progression and metastasis [41]. MSCs have also been shown to interact with tumor cells inducing chemoresistance, mediated by increased IL-6 levels and STAT signaling [37]. The effectiveness of immunotherapy is also impacted, as MSCs limit antibody-dependent cell cytotoxicity (ADCC) through TGFβ signaling [43]. Co-culture experiments of NK cells and tumor-associated neuroblastoma MSCs additionally revealed that non-senescent MSCs resisted the cytotoxic effects of NK cells [44]. On the contrary, MSCs have been shown to induce apoptosis and halt proliferation in neuroblastoma cells [45]. Finally, Schwann cells residing in the TME are generally thought to promote differentiation of neuroblastoma cells [46]. However, neuroblastoma cells express high levels of HGMB1, leading to Schwann cell autophagy through activation of TLR4 and ultimately proliferation of tumor cells [47]. Apart from cellular components of the TME, other components, such as stiffness of the extracellular matrix, hypoxia, extracellular vesicle transfer, and micro-RNAs, also contribute to neuroblastoma heterogeneity [29]. Cell populations and conditions of the microenvironment can significantly influence prognosis, survival, therapeutic options, and clinical implications in neuroblastoma.

## 3. Molecular Landscape of Neuroblastoma

The diversity of neuroblastoma in terms of clinical outcome, cellular composition, and establishment of the microenvironment is highly linked to the molecular biology of the disease. DNA ploidy has been linked to risk stratification in neuroblastoma, showcasing the association of whole chromosome losses and gains with a more favorable outcome, while tumors with segmental alterations are associated with poor patient survival. Statistically significant segmental alterations associated with poor outcome are 1q, 2p, 7q, 11q13.3, 12q, and 17q gains, as well as 1p, 3p, and 11q deletions [48,49,50,51] (Figure 1). While lower-stage tumors are usually hyperdiploid, high-risk cases frequently contain diploid tumor cells [52]. Among the primary high-risk cases, segmental gain of 17q is the most common aberration, characterizing 90% of tumor samples with gains larger or equal to the minimum region between 17q23.1 and 17qter [48,53,54,55]. Therefore, genes located in 17q, such as *PPM1D*, *BIRC5*, *IGF2BP1*, and others, are researched as potential initiators or therapeutic targets for neuroblastoma [54,55,56].

**Figure 2:**
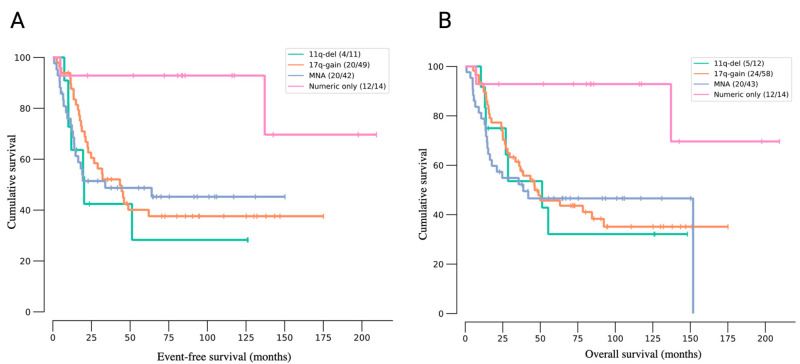
Kaplan–Meier plots illustrating the event-free (**A**) and overall (**B**) survival probabilities of neuroblastoma patients presenting common genomic rearrangements. The information for these plots was obtained from the “Tumor Neuroblastoma HR—de Preter” database available in R2 (https://hgserver1.amc.nl/cgi-bin/r2/main.cgi (accessed on 24 April 2024)). (**A**) Out of 556 patients in the database, 116 presented only one type rearrangement included in the figure (i.e., 11q deletion “11q-del”, 17q gain “17q-gain”, MYCN amplification “MNA”, and full chromosome changes “Numeric only”). A box within the figure includes, in parentheses, the patients censored and the total number of patients with the rearrangement. (**B**) Out of 556 patients in the database, 174 presented only one type rearrangement included in the figure (i.e., 11q deletion “11q-del”, 17q gain “17q-gain”, MYCN amplification “MNA”, and full chromosome changes “Numeric only”). A box within the figure includes, in parentheses, the patients censored and the total number of patients with the rearrangement.

The majority of neuroblastoma cases occur sporadically, whereas 1–2% of the cases are derived from germline mutations. Germline mutations in the anaplastic lymphoma kinase (*ALK*) gene are found in 80% of familial cases, while around 10% of neuroblastoma cases display somatic *ALK* mutations [57,58]. The paired-like homeobox 2B (*PHOX2B*) transcription factor is known to regulate early differentiation of adrenal chromaffin cells [59]. *PHOX2B* gene mutations are identified in 6% of familial cases and fewer sporadic cases [59,60]. Also, genes involved in neuritogenesis, including genes responsible for controlling proper Rho/Rac signaling during embryonal development, are frequently mutated (28%) in neuroblastoma [61,62]. One of the first oncogenes to be associated with a poor outcome in neuroblastoma was *MYCN*, appearing amplified in more than 25% of primary neuroblastoma tumors (Figure 2). Due to its proximal location to *ALK* on chromosome arm 2p, *MYCN* and *ALK* are often co-amplified [49,60,63]. High-risk neuroblastoma may also manifest with rearrangements in the 5p15.33 region, resulting in mutually exclusive *ATRX*-inactivating or *TERT*-activating alterations [49,64,65]. Additional mutations, such as those in Ras-pathway downstream effectors, including *FGFR1*, *TP53*, *LIN28*, *PTPRD*, *SHANK2*, *BRCA2*, *MAPK*, *LMO1*, and *ARID1A/1B*, have been identified at lower frequencies in neuroblastoma [3,6,49,57,66,67,68].

The tumor cell heterogeneity observed in neuroblastoma is mainly characterized by cells with an ADRN or MES phenotype. These cell subtypes have distinct molecular characteristics and super-enhancer profiles. Noradrenergic transcription factors are expressed in ADRN cells, including *PHOX2B*, *GATA3*, *HAND2*, *ISL1*, *TBX2*, and *ASCL1* [17,19,20,69]. MES cells, on the other hand, exhibit a more neural crest cell precursor identity. MES cells have been identified to express IRF1/IRF2/IRF3 and RUNX1/RUNX2, as well as PRRX1, and have been associated with NOTCH, YAP/TAZ, and RAS signaling pathways [19,20].

## 4. Current Treatments of Patients with Neuroblastoma

Neuroblastoma treatment protocols are highly dependent upon disease risk classification. For patients with low-risk tumors, observation is suggested for L1 and MS tumors lacking symptoms of the disease and for L2 tumors with favorable characteristics in patients younger than 18 months of age. For children older than 12 months with L1 tumors, however, gross total resection (GTR) might be necessary—especially for those with MYCN amplification followed by tumor observation. Biopsy sampling for risk determination for L2 and asymptomatic MS tumors is necessary in order to adapt the treatment strategy [6,70,71,72]. Intermediate-risk patient treatment is mainly response-based therapy and usually involves up to eight cycles of chemotherapy, followed by maximal excision of residual tumors in some patients [73]. Chemotherapy alone can reduce image-defined risk factors (IDRFs) in several patients with L2 disease [74,75], while it is the first step for treating MS patients, followed by biopsy sampling after stabilization. Certain intermediate-risk cases can be treated with additional differentiation therapy [6,73]. An overall survival of more than 5 years is accomplished in more than 95% of low-risk patients and more than 88% of intermediate-risk patients [76].

High-risk disease treatment is roughly divided into three stages: induction, consolidation, and maintenance therapy. Multiple clinical trials are aiming to improve the already established protocols, increasing patient survival and well-being. During induction, patients usually undergo five to eight cycles of chemotherapy according to the rapid COJAC protocol agreed upon by the SIOP European Neuroblastoma Group (SIOPEN) [77,78]. Patient stem cells are collected after the second (Children’s Oncology Group, COG) or eighth (SIOPEN) cycle of chemotherapy from the bone marrow or the periphery, and autologous stem cell transplantation (ASCT) occurs later, during the consolidation phase [6,78,79]. At the end of induction, maximal tumor excision can be attempted if morbidity is not risked [80]. In order to eradicate remaining traces of the disease in high-risk patients, high-dose chemotherapy is administered together with ASCT. Carboplatin, etoposide, and melphalan (CEM) is the chemotherapy regimen suggested by COG, while SIOPEN has utilized the less toxic busulfan/melphalan (Bu/Mel) [81,82]. Consolidation usually ends with post-recovery radiation therapy aimed at the primary tumor, as well as the metastatic sites [83]. Maintenance therapy includes isotretinoin (13-cis-retinoic acid), which induces differentiation of neuroblastoma tumor cells, together with immunotherapy, such as anti-disialoganglioside (anti-GD2) antibodies and cytokines (GM-CSF and IL-2) [84,85]. Maintenance therapy has been added to the guidelines as a means to help prevent relapses from residual disease.

## 5. Emerging Therapies

Despite continuous optimizations on current treatment protocols, neuroblastoma relapse and refractory disease are still hard to cure, and late-onset effects arise in patients treated with aggressive therapy [6,86]. While new diagnostic and prognostic markers such as cell-free DNA and nucleolin [87,88] are under investigation, novel therapeutic options have emerged and are under clinical evaluation or are gradually adopted in clinical practice. Immunotherapy and molecular targeting therapy are the pillars of these new therapeutic options. Since multiple signaling pathways have been associated with neuroblastoma pathogenesis, molecular therapeutics have mainly focused on inhibiting effectors of p53-MDM2, RAS-MAPK, and ALK signaling pathways. Additionally, research in targeting genetic and protein aberrations with small molecule inhibitors has led to the development of MYCN, PHOX2B, LIN28B, VEGF, BIRC5, and TrK inhibitors, while efforts on targeting epigenetic regulations pose another promising option [89]. Targeted therapy or precision medicine for neuroblastoma is a research area that has been given a lot of attention during the last decade and has been described in detail in numerous recent reviews [6,89,90,91,92,93,94,95].

## 6. The Immune Landscape of Neuroblastoma

Dissecting the immune landscape of neuroblastoma and detailed investigations of the cellular and molecular interactions between malignant and nonmalignant cells within the TME will be fundamental for designing possible immunotherapy strategies for neuroblastoma. Similarly to other cancers, neuroblastoma has evolved immune evasion strategies such as downregulation of MHC class-1 expression, increased activity of immune inhibitor factors such as TGFβ and arginase-2, and enhanced infiltration of suppressive myeloid cells [31,96,97,98]. In neuroblastoma, monocytic (M-MDSC) and polymorphonuclear myeloid-derived suppressor cells (PN-MDSCs) seem to be responsible for suppressing T-cell responses, while M-MDSC are more associated with neuroblastoma tumor progression compared to PN-MDSC [99,100].

The current knowledge regarding the presence and functions of neutrophils within the TME of neuroblastoma, their interactions with tumor cells, and their prognostic significance is still not understood [100]. Conflicting data have been reported on the correlation between the number of neutrophils and disease progression. Reports have concluded that increased neutrophil counts correlate to low-risk disease and good prognosis [101,102], whereas other reports showed no correlations [103,104] or were unable to detect neutrophils within the tumor samples [31] or show that increased tumor infiltration of neutrophils is correlated to poor survival. Adding to the inconsistency, neutrophils have been shown to have both anti- and pro-tumor effects in vitro and play an important role as effector cells in anti-GD2 immunotherapy [105,106].

Mast cells and basophils have not been found to be associated with neuroblastoma; however, eosinophils expressing insulin-like growth factor IGF-2 have been identified in tumors and linked to worse patient survival [100]. The majority of myeloid cells in neuroblastoma tumors are dendritic cells (DCs), monocytes, and macrophages [31,107]. Dendritic cells have been associated with a better prognosis [108]. In human neuroblastoma, the M2 macrophage phenotype is more common than M1, and M2 has been associated with worse clinical prognosis and metastasis to the bone marrow [100,109,110]. However, others discovered lower M2 levels in high-risk cases [31]. Lymphoid components of the neuroblastoma TME mainly include B, T, and NK cells. Active B cells, plasma cells, germinal center (GC) B cells, and memory B cells were identified in patient samples, with intermediate- and high-risk cases exhibiting elevated memory B cells and reduced GC B cells [31]. Cytotoxic NK cell numbers were also found to be elevated in patients of the same risk groups. Looking into T-cell populations, neuroblastoma shows infiltration of Tregs, CD4+ Th17, naïve, CD8+ cytotoxic T-cells, and NKT cells. Significant correlation of CD8+ cytotoxic T-cell clones, Th17, and naïve T-cell clones with improved survival have been reported [31].

The heterogeneity observed in neuroblastoma is also reflected in the immune landscape. Based on risk and MYCN amplification, Masih et al. identified distinct clusters depicting low-risk, MYCN-non-amplified high-risk, and MYCN-amplified ultra high-risk neuroblastoma cases [111]. While the ultra high-risk cases showed a generally cold TME, with few tumor-infiltrating lymphocytes (TILs) and downregulation of major histocompatibility complex (MHC) class II, the MYCN-non-amplified high-risk groups were characterized by a hot TME, exhibiting increased infiltration by NK and CD8+ T-cells and expression of immune checkpoint proteins. Additionally, there was a second MYCN-non-amplified high-risk clone with a higher stroma signature and an immunosuppressive TME, characterized by Treg, tumor-associated macrophages (TAMs), and MDSC populations [111].

## 7. Immunotherapeutic Approaches for Neuroblastoma

**Figure 3:**
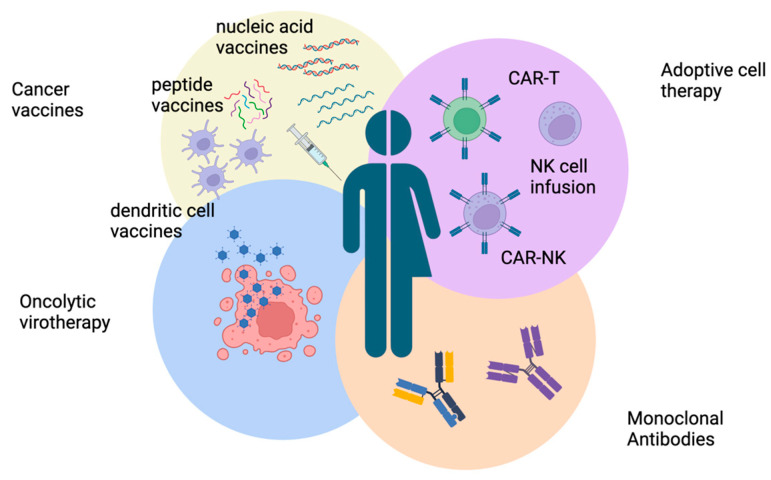
Overview of immunotherapy types developed for neuroblastoma. Immunotherapy options under research for neuroblastoma treatment include adoptive cell therapy, monoclonal antibodies, oncolytic virotherapy, and cancer vaccines. Created with https://www.biorender.com/ (accessed on 7 April 2024).

The incorporation of anti-GD2 antibody immunotherapy in neuroblastoma treatment protocols has been widely adopted. However, since the majority of neuroblastomas are regarded as immunologically cold tumors, they have evolved immune evasion mechanisms that could be harnessed for immunotherapy. Generating immune responses by engineering patient cells or viruses, as well as targeting multiple targets and stimulating the immune system, led to the development of neuroblastoma vaccines, monoclonal antibodies, oncolytic virotherapy, and adoptive cell therapy techniques, which have been actively researched in the last decades (Figure 3).

### 7.1. Neuroblastoma Vaccines

Achieving immunization using cancer vaccines is a growing field of immunotherapy. Types of cancer vaccines include peptide- and nucleic acid-based (DNA or RNA) vaccines, as well as vaccines based on viral vectors and cell-based vaccines, using mainly cancer and immune cells [112]. The majority of neuroblastoma vaccines that have been in clinical trials since 2000 are tumor or immune cell vaccines (Table 1). Dendritic cell (DC) vaccines are based on the capacity of DCs to present antigens to naive T-cells, eliciting an immune response targeted to a particular antigen. Autologous DC vaccines against neuroblastoma antigens have been included in clinical trials, including a phase II trial (NCT00405327) aiming to assess the efficacy of immune responses induced by a tumor lysate-pulsed DC vaccine administered post-transplantation of hematopoietic stem cells (HSCTs).

Allogeneic vaccines share significant similarities with autologous vaccines; however, instead of tumor antigens deriving from the patient, cancer cell lines expressing the specific tumor-associated antigens (TAAs) are used. Most commonly, cancer cell lines recognizing tumor-associated antigens (TAAs) unique to a particular tumor type are selected [113]. For instance, an ongoing trial is using non-modified SKNLP and modified SJNB-JF-IL2 and SJNB-JF-LTN neuroblastoma cell lines for vaccination of high-risk neuroblastoma patients (NCT00101309). Finally, GVAX is widely known for triggering immune activation and boosting antibody-dependent cell-mediated cytotoxicity (ADCC) owing to the release of Granulocyte–macrophage colony-stimulating factor (GM-CSF) [114]. This vaccine is generated by removing patient neuroblastoma cells, genetically modifying them to produce human GM-CSF, and delivering them as a vaccination against the tumor, together with immune checkpoint blockade (NCT04239040).

DNA and peptide vaccines have also been developed for neuroblastoma, using different platforms. Salmonella-based therapy uses Salmonella strains as platforms for cancer vaccines. In a recent phase I trial (NCT04049864), S. Typhimurium SS2017 carrying DNA plasmids and expressing TAAs for neuroblastoma was administered as a DNA vaccine in patients with relapsed neuroblastoma [115]. TAAs in this study were selected from tyrosine hydroxylase (TH), Phox2B, Survivin, MAGEA1, MAGEA3, and PRAME, based on the highest expression in the patient tumor biopsy sample. Finally, multiple trials administering a bivalent vaccine using neuroblastoma-associated antigens GD2 and GD3, conjugated to keyhole limpet hemocyanin (KLH) protein, have been set up (NCT00911560, NCT04936529, NCT06057948), testing this vaccine in combination with OPT-821 adjuvant and β-glycan [116]. Other vaccines include Racotumomab for N-glycolyl GM3-positive neuroblastoma, currently in a phase II clinical trial (NCT02998983), and a DNA vaccine encoding Galectin-1-derived peptide epitopes [117] in the preclinical testing stage.

**Table 1:** Clinical trials using cancer vaccine options for the treatment of neuroblastoma.

NCT Identifier	Study Title	Status/Outcome
NCT06057948	A Study of a Vaccine in Combination With Beta-glucan in People With Neuroblastoma	Recruiting
NCT00048386	Neuroblastoma Vaccine for Treatment of High-Risk Neuroblastoma After Chemotherapy	Completed; no results posted
NCT01192555	Allogeneic Tumor Cell Vaccination With Oral Metronomic Cytoxan in Patients With High-Risk Neuroblastoma	Active, not recruiting
NCT00405327	A Pilot Study of Tumor Cell Vaccine for High-risk Solid Tumor Patients Following Stem Cell Transplantation	Completed; no results posted
NCT00911560	Bivalent Vaccine With Escalating Doses of the Immunological Adjuvant OPT-821, in Combination With Oral Œ≤-glucan for High-Risk Neuroblastoma	Active, not recruiting
NCT04936529	A Study of a Vaccine in Combination With Œ≤-glucan and GM-CSF in People With Neuroblastoma	Recruiting
NCT01241162	Decitabine Followed by a Cancer Antigen Vaccine for Patients With Neuroblastoma and Sarcoma	Completed; vaccine tolerance and feasibility were indicated for patients with relapsed solid tumors [118]
NCT04239040	GVAX Plus Checkpoint Blockade in Neuroblastoma	Recruiting
NCT00703222	A Phase I/II Study Of Immunization With Lymphotactin And Interleukin 2 Gene Modified Neuroblastoma Tumor Cells (CHESAT)	Active, not recruiting
NCT01953900	iC9-GD2-CAR-VZV-CTLs/Refractory or Metastatic GD2-positive Sarcoma and Neuroblastoma (VEGAS)	Active, not recruiting
NCT00101309	Vaccine Therapy and Interleukin-2 in Treating Young Patients With Relapsed or Refractory Ewing’s Sarcoma or Neuroblastoma	Unknown
NCT04049864	DNA Vaccination Against Neuroblastoma	Unknown
NCT02998983	Racotumomab in Patients With High-risk Neuroblastoma	Completed; no results posted

### 7.2. Monoclonal Antibodies

Applying monoclonal antibodies as treatment modalities for pediatric cancer has increased during the last decades. For neuroblastoma, three anti-GD2 antibodies have been approved for patient treatment, based on the uniform expression of GD2 antigen on neuroblasts, aiding the attachment, invasion, and proliferation of cancer cells [119]. NK-induced antibody-dependent cellular cytotoxicity (ADCC) is the proposed mechanism of action for anti-GD2 antibodies in neuroblastoma immunotherapy [120]. Murine 3F8 is an anti-GD2 IgG3 antibody, tested alone or in combination with GM-CSF and β-glucan (NCT00492167). Since many patients developed human anti-mouse antibodies (HAMAs) [119,121,122,123], an IgG1 humanized version of this antibody called Naxitamab was developed (Hu3F8) [124] and was approved for patients with refractory or relapsed high-risk neuroblastoma in combination with GM-CSF [125] after successful clinical trial results (NCT03363373, NCT01757626). Similarly, murine 14G2a, which caused patients HAMA production with or without IL-2 [126,127], led to the development of chimeric mouse/human antibody Dinutuximab or Ch14.18. Ch14.18 was tested alone or in combination with CM-CSF and IL-2 [128,129]. Multiple clinical trials applying Dinutuximab in combination with other treatments have been performed (Table 2). Dinutuximab-beta (ch14.18/CHO) reports biosimilarity to Dinutuximab and is currently being evaluated in clinical trials, as is humanized Hu14.18K322A recombinant antibody [119]. Because of the K322A point mutation, treatment with the Hu14.18K322A antibody was experienced as less painful by patients compared to other anti-GD2 antibodies [130]. The addition of Hu14.18K322A to induction chemotherapy was recently identified to halt progression, while patients exhibited an 85.7% (95% CI, 70.9–93.3) 2-year event-free survival [131]. Finally, Hu3F8-BsAb Nivatrotamab bispecific antibody was tested in relapsed/refractory NB patients, measuring dose-limiting toxicities in a clinical trial (NCT03860207). Bispecific antibodies, having two binding sites, could represent the future of monoclonal antibody immunotherapy [132].

Apart from GD2, antibodies targeting other proteins overexpressed in neuroblastoma have been developed. Such antibodies include the ones recognizing the transmembrane glycoprotein B7-H3, which participates in NK and T-cell function [133,134]. Omburtamab (8H9) and Enoblituzumab represent murine and humanized antibodies targeting B7-H3 respectively. Recently, a phase I clinical trial (NCT02982941) was initiated to evaluate Enoblituzumab effect in neuroblastoma and other solid tumors. Ultimately, the antibodies tested preclinically targeted O-acetyl GD2, ALK, PD-1, CD47, and GPC2 [119]. Neuroblastoma is a tumor harboring a few genetic mutations limiting the use of neoantigen-based therapies. However, a recent study investigating the immunopeptidome of neuroblastoma shows that a QYNPIRTTF discovered on HLA-A*24:02, deriving from the master transcription regulator PHOX2B, can be targeted by constructing peptide-centric chimeric antigen receptors (PC-CARs) recognizing the PHOX2B-derived peptide. Treatment of mice carrying established neuroblastoma with PC-CAR T-cells resulted in complete tumor regression. Together, these suggest that the immunotherapeutic treatment options can be expanded using PC-CAR in tumors exhibiting low expression of surface neo-antigens [135].

**Table 2:** Clinical trials using monoclonal and conjugated antibodies for the treatment of neuroblastoma.

NCT Identifier	Study Title	Status/Outcome
NCT04909515	Naxitamab and Granulocyte–Macrophage Colony Stimulating Factor (GMCSF) and Isotretinoin for Consolidation of Patients With High-Risk Neuroblastoma in First Remission.	Withdrawn
NCT05489887	Naxitamab Added to Induction for Newly Diagnosed High-Risk Neuroblastoma	Recruiting
NCT04560166	Naxitamab and GM-CSF in Combination With IT in Patients With High-Risk Neuroblastoma	Terminated (due to business priorities)
NCT06013618	Clinical Analysis of Naxitamab (hu3F8) in the Treatment of Pediatric High Risk or Refractory/Relapsed Neuroblastoma	Recruiting
NCT03363373	Naxitamab for High-Risk Neuroblastoma Patients With Primary Refractory Disease or Incomplete Response to Salvage Treatment in Bone and/or Bone Marrow	Recruiting
NCT04501757	Naxitamab and GM-CSF in People With Neuroblastoma	No longer available
NCT06047535	Naxitamab and Granulocyte–Macrophage Colony Stimulating Factor (GM-CSF) Combined With Isotretinoin for Maintenance Treatment of Patients With High-Risk Neuroblastoma in First Complete Response.	Not yet recruiting
NCT02308527	Activity Study of Bevacizumab With Temozolomide ¬± Irinotecan for Neuroblastoma in Children	Active, not recruiting
NCT02693171	Post-Marketing Assessment of Immunogenicity and Safety of UnituxinÆ in High-Risk Neuroblastoma Patients	Terminated (sponsor decision)
NCT05272371	Immunotherapy With Dinutuximab Beta in Combination With Chemotherapy for the Treatment of Patients With Primary Neuroblastoma Refractory to Standard Therapy and With Relapsed or Progressive Disease	Recruiting
NCT01701479	Long-Term Continuous Infusion ch14.18/CHO Plus s.c. Aldesleukin (IL-2)	Unknown
NCT03794349	Irinotecan Hydrochloride, Temozolomide, and Dinutuximab With or Without Eflornithine in Treating Patients With Relapsed or Refractory Neuroblastoma	Active, not recruiting
NCT02914405	Phase I Study of 131-I mIBG Followed by Nivolumab and Dinutuximab Beta Antibodies in Children With Relapsed/Refractory Neuroblastoma	Recruiting
NCT02169609	Safety Study of Dinutuximab Combined With Immunotherapy to Treat Neuroblastoma	Completed; no results posted
NCT01711554	Lenalidomide and Dinutuximab With or Without Isotretinoin in Treating Younger Patients With Refractory or Recurrent Neuroblastoma	Active, not recruiting
NCT05400603	Allogeneic Expanded Gamma Delta T-cells With GD2 Chemoimmunotherapy in Relapsed or Refractory Neuroblastoma	Recruiting
NCT02743429	Phase II Study of Monoclonal Antibody ch14.18/CHO Continuous Infusion in Patients With Primary Refractory or Relapsed Neuroblastoma	Active, not recruiting
NCT05373901	Evaluation of the Safety and Pharmacokinetics of Dinutuximab Beta as Maintenance Therapy in Chinese Patients With High-risk Neuroblastoma	Completed; no results posted
NCT02573896	Immunotherapy of Relapsed Refractory Neuroblastoma With Expanded NK Cells	Active, not recruiting
NCT06172296	Dinutuximab With Chemotherapy, Surgery, and Stem Cell Transplantation for the Treatment of Children With Newly Diagnosed High-Risk Neuroblastoma	Not yet recruiting
NCT01704716	High-Risk Neuroblastoma Study 1.8 of SIOP-Europe (SIOPEN)	Recruiting
NCT01592045	ch14.18 Pharmacokinetic Study in High-Risk Neuroblastoma	Completed; pharmacokinetics results can be accessed on clinicaltrials.org
NCT04211675	NK Cells Infusions With Irinotecan, Temozolomide, and Dinutuximab	Recruiting
NCT03126916	Testing the Addition of 131I-MIBG or Lorlatinib to Intensive Therapy in People With High-Risk Neuroblastoma (NBL)	Active, not recruiting
NCT02641782	NB2013-HR German (GPOH)/Dutch (DCOG) Trial	Terminated
NCT02258815	CH14.18 1021 Antibody and IL2 After Haplo SCT in Children With Relapsed Neuroblastoma	Completed; feasible treatment, with low chance of graft-versus-host disease [136]
NCT04253015	A Post-Authorization Safety Study Patient Registry of Patients With Neuroblastoma Being Treated With Dinutuximab Beta	Recruiting
NCT01041638	Monoclonal Antibody Ch14.18, Sargramostim, Aldesleukin, and Isotretinoin After Autologous Stem Cell Transplant in Treating Patients With Neuroblastoma	Completed; identified toxicities from treatment and potential biomarkers [137]
NCT04385277	Treatment With Dinutuximab, Sargramostim (GM-CSF), and Isotretinoin in Combination With Irinotecan and Temozolomide After Intensive Therapy for People With High-Risk Neuroblastoma (NBL)	Active, not recruiting
NCT01767194	Irinotecan Hydrochloride and Temozolomide With Temsirolimus or Dinutuximab in Treating Younger Patients With Refractory or Relapsed Neuroblastoma	Completed; patients with relapsed/refractory disease exhibited significant antitumor response [138]
NCT03786783	Dinutuximab, Sargramostim, and Combination Chemotherapy in Treating Patients With Newly Diagnosed High-Risk Neuroblastoma	Active, not recruiting
NCT04751383	Testing the Combination of Two Immunotherapy Drugs (Magrolimab and Dinutuximab) in Patients With Relapsed or Refractory Neuroblastoma or Relapsed Osteosarcoma	Suspended (unacceptable toxicity)
NCT03332667	MIBG With Dinutuximab +/− Vorinostat	Active, not recruiting
NCT05421897	Rapid Administration Pilot for Infusing Dinutuximab	Recruiting
NCT06071897	Induction Chemoimmunotherapy for Patients With High-Risk Neuroblastoma	Recruiting
NCT04238819	A Study of Abemaciclib (LY2835219) in Combination With Other Anti-Cancer Treatments in Children and Young Adult Participants With Solid Tumors, Including Neuroblastoma	Recruiting
NCT00030719	Combination Chemotherapy With or Without Filgrastim Before Surgery, High-Dose Chemotherapy, and Radiation Therapy Followed by Isotretinoin With or Without Monoclonal Antibody in Treating Patients With Neuroblastoma	Unknown
NCT01857934	Therapy for Children With Advanced Stage Neuroblastoma	Active, not recruiting
NCT05608148	Clinical Trial of GAIA-102 for Refractory/Relapse Neuroblastomas or Malignant Pediatric Solid Tumors With Lung Metastases	Recruiting
NCT00026312	Isotretinoin With or Without Dinutuximab, Aldesleukin, and Sargramostim Following Stem Cell Transplant in Treating Patients With Neuroblastoma	Active, not recruiting
NCT01704872	ch14.18/CHO Bridging Study	Completed; similar side effects as observed in ch14.18/SP2/0 studies; treatment accepted for further evaluation [139]
NCT05754684	Quadruple Immunotherapy for Neuroblastoma	Recruiting
NCT02559778	Pediatric Precision Laboratory Advanced Neuroblastoma Therapy	Recruiting
NCT00005576	Monoclonal Antibody Therapy With Sargramostim and Interleukin-2 in Treating Children With Neuroblastoma	Completed; no results posted
NCT04221035	High-Risk Neuroblastoma Study 2 of SIOP-Europa-Neuroblastoma (SIOPEN)	Recruiting
NCT01701479	Long-Term Continuous Infusion ch14.18/CHO Plus s.c. Aldesleukin (IL-2)	Unknown
NCT02914405	Phase I Study of 131-I mIBG Followed by Nivolumab and Dinutuximab Beta Antibodies in Children With Relapsed/Refractory Neuroblastoma	Recruiting
NCT02743429	Phase II Study of Monoclonal Antibody ch14.18/CHO Continuous Infusion in Patients With Primary Refractory or Relapsed Neuroblastoma	Active, not recruiting
NCT01704716	High-Risk Neuroblastoma Study 1.8 of SIOP-Europe (SIOPEN)	Recruiting
NCT02258815	CH14.18 1021 Antibody and IL2 After Haplo SCT in Children With Relapsed Neuroblastoma	Completed; the antibody dosing regimen was adequate and the lymphoid immune compartment exhibited strong performance [140]
NCT00743496	A Phase I Trial Of The Humanized Anti-GD2 Antibody In Children And Adolescents With Neuroblastoma, Osteosarcoma, Ewing Sarcoma, and Melanoma	Completed; pre-existing antitherapeutic antibodies may be associated with increased antitumor effects [141]
NCT02130869	A Pilot Study of Immunotherapy Including Haploidentical NK Cell Infusion Following CD133+ Positively Selected Autologous Hematopoietic Stem Cells in Children With High-Risk Solid Tumors or Lymphomas	Completed; no results posted
NCT02159443	Pretreatment Anti-Therapeutic Antibodies (PATA) in Patients Treated With hu14.18K322A Antibody	Completed; no results posted
NCT01576692	Combination Chemotherapy, Monoclonal Antibody, and Natural Killer Cells in Treating Young Patients With Recurrent or Refractory Neuroblastoma	Completed; combination therapy was tolerable, safe, and feasible for patients with relapsed/refractory neuroblastoma and exhibits promising antitumor effects [142]
NCT01857934	Therapy for Children With Advanced Stage Neuroblastoma	Active, not recruiting
NCT00582608	Tumor Detection Using Iodine-131-Labeled Monoclonal Antibody 8H9	Terminated
NCT00089245	Radiolabeled MAB Therapy in Patients With Refractory, Recurrent, or Advanced CNS or Leptomeningeal Cancer	Terminated

### 7.3. Oncolytic Virotherapy

Oncolytic virotherapy is a form of immunotherapy that induces the lysis of tumor cells through the use of natural or genetically modified viruses. Oncolytic viral (OV) action includes infection of tumor cells and subsequent oncolysis inducing anti-tumoral and anti-viral immunity. On the one hand, antigens from the lysed tumor cells released into the extracellular space may be presented to lymphocytes by dendritic cells and activate anti-cancer immune responses, while on the other hand, anti-viral components of the TME may target the viruses in order to clear the viral infection. Additionally, engineered OVs with genes encoding cytokines such as GM-CSF, IL-2, and others have been applied in order to boost the immune response [143,144].

Oncolytic virotherapy against neuroblastoma has been attempted in clinical (Table 3) and preclinical testing, mainly using double-stranded DNA viruses, including adenovirus, vaccinia virus, and HSV, as well as the RNA Seneca valley virus [143]. Regarding adenovirus-based oncolytic virotherapy, Celyvir consists of autologous marrow-derived mesenchymal stem cells (MSCs) carrying ICOVIR-5, a novel oncolytic adenovirus derived from AdΔ24RGD, controlled by the E2F promoter. This adenovirus selectively replicates in cancer cells by activating the Rb/E2F pathway [145]. The role of MSCs is to shield the oncolytic virus from immune components in the bloodstream, allowing the virus to reach metastatic sites and achieve a targeted therapeutic effect [146]. In a recent clinical trial (NCT01844661), Celyvir demonstrated significant therapeutic efficacy, establishing its safety and justifying further evaluation in a phase II setting [147]. Genetically engineered adenoviruses, including a ZD55 adenovirus carrying shMYCN RNA, as well as OBP-301 and OBP-702 adenoviruses driven by the hTERT promoter, showed promising results when tested on neuroblastoma cell lines and xenografts [143,148,149,150]. Furthermore, the modified vaccinia virus Pexa-Vec (JX-594) was generated by removing the thymidine kinase gene and introducing both the GM-CSF gene and lac-Z gene into the Wyeth vaccine strain. Oncolytic virotherapy phase I trial NCT01169584 applied Pexa-Vec on two pre-treated patients, proving the safety of intratumoral injection using this virus and paving the way for further clinical testing [151]. VV-GD2m-NAP, another genetically engineered vaccinia virus, was synthesized by incorporating into the Western Reserve (WR) strain the genes for neutrophil-activating protein (NAP) and disialoganglioside mimotope (GD2m). When tested on subcutaneously injected NSX2 neuroblastoma xenografts, tumor growth was controlled and mouse survival was prolonged [152]. Additionally, the phase I trial of the Seneca Valley Virus NTX-010 (NCT01048892) showed it was safely tolerated by children with neuroblastoma. However, further validation of its treatment effects is necessary [153]. Finally, preclinical testing of many developed Herpes simplex viruses (HSVs) shows promising results for neuroblastoma immunotherapy, as reviewed elsewhere [143].

### 7.4. Adoptive Cell Therapy

Adoptive cell therapy (ACT) has become the most well-known type of immunotherapy owing to chimeric antigen receptor T-cell therapy (CAR-T) and its successful therapeutic results. Importantly, the quiver of APC includes more options than CAR-T cells: tumor-infiltrating lymphocyte therapy (TIL), engineered T-cell receptor cell therapy (TCR)-T, and NK and NKT cell infusion, as well as other CAR techniques, such as CAR-natural killer cells (CAR-NK), CAR-natural killer cell T-cells (CAR-NKT), and CAR-γδT and CAR-macrophages (CAR-M). Of these, NK and NKT cell infusion, as well as CAR techniques, have been the focus of APC therapy in neuroblastoma.

NK cell infusion has shown promise in anti-cancer immunotherapy treatment based on the ability of NK cells to secrete cytolytic granules, cytokines, and chemokines and activate other immune cell types and ADCC. Low NK infiltration is identified in a number of tumor types; therefore, infusion of NK cells in combination with other treatments has been widely adopted in multiple clinical trials [155]. Since neuroblastoma lacks HLA-class I expression, NK cells that target non-HLA class I-expressing cells could significantly increase the therapeutic effects of immunotherapy. Expansion of NK cells can be achieved via cytokine cocultures and/or feeder cells like K562 [156]. In the preclinical context, NK cell infusion was tested on SCID/NOD mouse xenografts generated using HTLA-230 [157] and CHLA-255-Fluc neuroblastoma cells [158]. In the metastatic model, increased survival and lower BM infiltration were observed after infusion with IL-2 and IL-15-activated NK cells [157]. The second model treatment with NK cells expanded by co-culture with K562 mbIL-21 cells and IL-2 led to prolonged survival when administered in combination with the anti-GD2 ch14.18 antibody [158]. Combination therapy with the addition of NK cell infusion has been adopted in clinical trials. NK cells are obtained from donors being either allogeneic or haploidentical, with various techniques having been outlined for in vitro expansion of NK cells [159]. Following leukapheresis and purification using the CliniMACS system, GMP-graded NK cell infusion was proven to be well tolerated, non-toxic, and safe in combination with anti-GD2 Ab hu14.18K322A, GM-CSF, and IL2 for patients pre-treated with chemotherapy [142,160]. A phase I clinical trial of patients receiving different dosages of NK cells together with anti-GD2 murine 3F8 antibody additionally showed that patients receiving a higher NK cell dose had a higher progression-free survival [161]. A number of clinical trials have been registered applying combination therapy together with NK cell infusion to neuroblastoma patients (Table 4).

Genetically engineered T-cells have been very successful in the treatment of hematologic cancers. CAR T-cells are synthesized using an intracellular signal transduction domain fused to a transmembrane spacer (hinge domain) and a single-chain variable fragment (scFv). Thanks to the extracellular scFv, antigen recognition takes place. The variable regions of the two immunoglobulin chains of a specific epitope-targeting antibody are fused together and connected by a short linker peptide, ultimately forming the scFv. The spacer or hinge domain is the link between the scFv domain and the T-cell membrane [162]. Five generations of CAR T-cells have been developed so far, distinguished by the elements within the intracellular signaling domain. These progress from basic TCR complex ζ chains (first generation) to include CD137 and CD28 co-stimulatory molecules (second and third generation) and regions inducing IL-12 (fourth generation), finally incorporating receptors specific for STAT-3/5 (fifth generation). The objective of the fifth-generation CAR T-cells is to eradicate even cancer cells that do not express the targeted antigen locally and foster the development of memory T-cells [163,164,165].

CAR T-cell therapy for neuroblastoma has mostly been focused on GD2 antigen, and several ongoing clinical trials are investigating combination therapy with anti-GD2 CAR T-cell therapy (Table 4). In an ongoing clinical trial (NCT03721068), GD2 CAR T-cells were engineered to express IL-15 together with inducible caspase 9. Moreover, another ongoing trial is studying GD2 CAR T-cells expressing a constitutively active IL-7 receptor for the treatment of relapsed and/or refractory neuroblastoma (NCT03635632). Lastly, CAR T-cells targeting B7-H3 (NCT04483778) and L1CAM (NCT02311621) are also being investigated in clinical trials.

Recently, NKT cells have emerged as a potential anti-tumor therapy for neuroblastoma. NKT cells are a subgroup of T-cells co-expressing NK cell markers. They recognize lipids and glycolipids presented via CD1d, an MHC class I-like molecule [166]. Preclinical research suggests that NKT cells transduced with the IL-15 gene could be a future adoptive cell therapy approach for neuroblastoma patients [159]. The first phase I clinical trial for the treatment of relapsed or refractory NB patients using autologous NKTs co-expressing a GD2-specific CAR with interleukin 15 (IL15) (GD2-CAR.15) is ongoing (NCT03294954). Preliminary results concluded the safety of GD2-CAR.15 NKTs and a 25% objective response rate [167]. Current immunotherapies included in clinical trials for the treatment of neuroblastoma are summarized in Figure 3.

**Table 4:** Clinical trials using adoptive cell therapy options for the treatment of neuroblastoma.

NCT Identifier	Study Title	Status/Outcome
NCT02761915	A Phase I Trial of Anti-GD2 T-cells (1RG-CART)	Completed; CAR T-cell therapy appears safe, with no on-target off-tumor toxicity. However, two patients exhibited cytokine release syndrome [168].
NCT02107963	A Phase I Trial of T-cells Expressing an Anti-GD2 Chimeric Antigen Receptor in Children and Young Adults With GD2+ Solid Tumors	Completed; feasibility and safety of administration was identified [169]
NCT05650749	GPC2 CAR T-cells for Relapsed or Refractory Neuroblastoma	Recruiting
NCT03721068	Study of CAR T-cells Targeting the GD2 With IL-15+iCaspase9 for Relapsed/Refractory Neuroblastoma or Relapsed/Refractory Osteosarcoma	Recruiting
NCT01822652	Third-Generation GD-2 Chimeric Antigen Receptor and iCaspase Suicide Safety Switch, Neuroblastoma, GRAIN	Active, not recruiting
NCT03373097	Anti-GD2 CAR T-cells in Pediatric Patients Affected by High Risk and/or Relapsed/Refractory Neuroblastoma or Other GD2-positive Solid Tumors	Recruiting
NCT03294954	GD2 Specific CAR and Interleukin-15 Expressing Autologous NKT Cells to Treat Children With Neuroblastoma	Recruiting
NCT04637503	4SCAR-T Therapy Targeting GD2, PSMA, and CD276 for Treating Neuroblastoma	Unknown
NCT01953900	iC9-GD2-CAR-VZV-CTLs/Refractory or Metastatic GD2-positive Sarcoma and Neuroblastoma	Active, not recruiting
NCT02439788	Third-Generation GD2-specific Chimeric Antigen Receptor Transduced Autologous Natural Killer T-cells for Neuroblastoma	Withdrawn
NCT04897321	B7-H3-Specific Chimeric Antigen Receptor Autologous T-cell Therapy for Pediatric Patients With Solid Tumors (3CAR)	Recruiting
NCT02765243	Anti-GD2 Fourth-Generation CAR T-cells Targeting Refractory and/or Recurrent Neuroblastoma	Completed; this therapy exhibited antitumor response without serious toxicities [170]
NCT04539366	Testing a New Immune Cell Therapy, GD2-Targeted Modified T-cells (GD2CART), in Children, Adolescents, and Young Adults With Relapsed/Refractory Osteosarcoma and Neuroblastoma, the GD2-CAR PERSIST Trial	Suspended
NCT05990751	Multi-modular Chimeric Antigen Receptor Targeting GD2 in Neuroblastoma	Not yet recruiting
NCT00085930	Blood T-cells and EBV Specific CTLs Expressing GD-2 Specific Chimeric T-cell Receptors to Neuroblastoma Patients	Active, not recruiting
NCT02311621	Engineered Neuroblastoma Cellular Immunotherapy (ENCIT)-01	Active, not recruiting
NCT04864821	Clinical Study of CD276 Targeted Autologous Chimeric Antigen Receptor T-cell Infusion in Patients With CD276-Positive Advanced Solid Tumor	Unknown
NCT02919046	Study Evaluating Efficacy and Safety With CAR-T for Relapsed or Refractory Neuroblastoma in Children	Unknown
NCT03635632	C7R-GD2.CAR T-cells for Patients With Relapsed or Refractory Neuroblastoma and Other GD2-Positive Cancers (GAIL-N)	Active, not recruiting
NCT04483778	B7H3 CAR T-cell Immunotherapy for Recurrent/Refractory Solid Tumors in Children and Young Adults	Active, not recruiting
NCT05562024	TAA06 Injection in the Treatment of Patients With B7-H3-Positive Relapsed/Refractory Neuroblastoma	Recruiting
NCT03618381	EGFR806 CAR T-cell Immunotherapy for Recurrent/Refractory Solid Tumors in Children and Young Adults	Recruiting
NCT02457650	T-cell Receptor-transduced T-cells Targeting NY-ESO-1 for Treatment of Patients With NY-ESO-1-Expressing Malignancies	Unknown
NCT05296564	Anti-NY-ESO-1 TCR Gene Engineered Lymphocytes Given by Infusion to Patients With NY-ESO-1-Expressing Metastatic Cancers	Recruiting
NCT02508038	Alpha/Beta CD19+ Depleted Haploidentical Transplantation + Zometa for Pediatric Hematologic Malignancies and Solid Tumors	Recruiting
NCT00085930	Blood T-cells and EBV-Specific CTLs Expressing GD-2 Specific Chimeric T-cell Receptors to Neuroblastoma Patients	Active, not recruiting
NCT00874315	Allogeneic Hematopoietic Stem Cell Transplantation for Relapsed or Refractory High-Risk NBL.	Withdrawn
NCT01807468	Haploidentical Stem Cell Transplantation and NK Cell Therapy in Patients With High-risk Solid Tumors	Unknown
NCT04211675	NK Cell Infusions With Irinotecan, Temozolomide, and Dinutuximab	Recruiting
NCT01287104	A Phase I Study of NK Cell Infusion Following Allogeneic Peripheral Blood Stem Cell Transplantation From Related or Matched Unrelated Donors in Pediatric Patients With Solid Tumors and Leukemias	Completed; while killing efficacy and receptor expression activation was significant, five out of nine participants demonstrated severe graft-versus-host disease [171]
NCT00698009	Haploidentical Natural Killer (NK) Cells in Patients With Relapsed or Refractory Neuroblastoma	Terminated
NCT02100891	Phase 2 STIR Trial: Haploidentical Transplant and Donor Natural Killer Cells for Solid Tumors	Completed; this treatment was well-tolerated, and overall survival was improved [172]
NCT01156350	Haploidentical Hematopoietic Stem Cell Transplantation Following Reduced-intensity Conditioning in Children With Neuroblastoma	Unknown
NCT03242603	Immunotherapy of Neuroblastoma Patients Using a Combination of Anti-GD2 and NK Cells	Unknown
NCT02650648	Humanized Anti-GD2 Antibody Hu3F8 and Allogeneic Natural Killer Cells for High-Risk Neuroblastoma	Active, not recruiting
NCT00788125	Dasatinib, Ifosfamide, Carboplatin, and Etoposide in Treating Young Patients With Metastatic or Recurrent Malignant Solid Tumors	Terminated
NCT01386619	NK DLI in Patients After Human Leukocyte Antigen (HLA)-Haploidentical Hematopoietic Stem Cell Transplantation (HSCT)	Completed; NKG2D-mediated cytotoxicity was repaired after haploidentical NK-DLI treatment [173]
NCT02573896	Immunotherapy of Relapsed Refractory Neuroblastoma With Expanded NK Cells	Active, not recruiting
NCT05754684	Quadruple Immunotherapy for Neuroblastoma	Recruiting
NCT00877110	Anti-GD2 3F8 Antibody and Allogeneic Natural Killer Cells for High-Risk Neuroblastoma	Completed; observed antitumor effect after activation of NK cells. Follow up study: NCT02650648 [161]
NCT01857934	Therapy for Children With Advanced Stage Neuroblastoma	Active, not recruiting
NCT01875601	NK White Blood Cells and Interleukin in Children and Young Adults With Advanced Solid Tumors	Completed; feasibility, safety, and tolerance of this strategy were observed [174]
NCT03209869	Treatment of Relapsed or Refractory Neuroblastoma and Osteosarcoma With Expanded Haploidentical NK Cells and Hu14.18-IL2	Withdrawn
NCT02130869	A Pilot Study of Immunotherapy Including Haploidentical NK Cell Infusion Following CD133+ Positively Selected Autologous Hematopoietic Stem Cells in Children With High-Risk Solid Tumors or Lymphomas	Completed; no results posted

## 8. Conclusions

The rapid advancement of tools for detailed analysis of single cells within tumor samples has enhanced our knowledge of the cellular and molecular landscape in neuroblastoma. This has given us valuable information regarding neuroblastoma heterogeneity and tumor cell plasticity, as well as clues regarding tumor cell evolution and cell of origin. Dissecting the cellular and molecular interactions of cells within the neuroblastoma microenvironment has given indications for potential new treatment modalities including anti-inflammatory and differentiation therapies. Furthermore, mapping of the immune cell landscape of neuroblastoma has also identified new potential therapy options which, in addition to the promising preclinical data obtained on CAR-T and PC-CAR-T cell therapies, give hope for curing more patients with high-risk neuroblastoma. However, the survival of patients experiencing refractory or relapsed neuroblastoma is still dismal, and more research, including the development of relevant preclinical models, needs to be employed to increase survival for this group of patients.

## Figures and Tables

**Table 3:** Clinical trials using oncolytic virotherapy options for the treatment of neuroblastoma.

NCT Identifier	Study Title	Status/Outcome
NCT01953900	iC9-GD2-CAR-VZV-CTLs/Refractory or Metastatic GD2-positive Sarcoma and Neuroblastoma	Active, not recruiting
NCT01048892	Seneca Valley Virus-001 and Cyclophosphamide in Treating Young Patients With Relapsed or Refractory Neuroblastoma, Rhabdomyosarcoma, or Rare Tumors With Neuroendocrine Features	Completed; tolerance and feasibility of NTX-010 with or without CTX was observed [153]
NCT01460901	Study of Donor-Derived, Multi-virus-specific, Cytotoxic T-Lymphocytes for Relapsed/Refractory Neuroblastoma	Completed; all three patients died of the disease and response was non-complete [154]
NCT00314925	Safety Study of Seneca Valley Virus in Patients With Solid Tumors With Neuroendocrine Features	Unknown
NCT05593328	Study of Onvansertib in Combination With FOLFIRI and Bevacizumab Versus FOLFIRI and Bevacizumab for Second-Line Treatment of Metastatic Colorectal Cancer in Participants With a Kirsten Rat Sarcoma Virus Gene (KRAS) or Neuroblastoma-RAS (NRAS) Mutation	Active, not recruiting
NCT01169584	Safety Study of Recombinant Vaccinia Virus to Treat Refractory Solid Tumors in Pediatric Patients	Completed; evaluated safety of administration to children, with no serious adverse effects [151]
